# Type Va extrahepatic bile duct duplication: a case report

**DOI:** 10.1186/s13256-019-2259-5

**Published:** 2019-10-28

**Authors:** Bharat Mani Banjade, Ashish Rajbhandari, Rabin Koirala, Tuhin Shah, Chitra Lal Bhattachan

**Affiliations:** Department of GI Surgery, Nepal Medical College & Teaching Hospital, Kathmandu, Nepal

**Keywords:** Bile duct anomaly, Extrahepatic bile duct duplication, Accessory bile duct, Double bile ducts

## Abstract

**Background:**

Extrahepatic bile duct duplication is an extremely rare congenital anomaly in which two common bile ducts exist. There are five different types of this anomaly and we present an unusual variant of duplication of an extrahepatic biliary system of type Va variety.

**Case presentation:**

This case report describes a 63-year-old women from rural Nepal who presented with type Va of duplicated extrahepatic bile duct, with chronic calculous cholecystitis and choledocholithiasis. She was managed with cholecystectomy with hepatic ductoplasty and hepaticojejunostomy.

**Conclusion:**

A rare case of double common bile duct (type Va) complicated by choledocholithiasis, cholangitis, and chronic cholecystitis is reported here. Rare cases are sometimes overlooked by modern diagnostic techniques. Correct diagnosis helps appropriate surgical intervention.

## Background

**Fig. 1:**
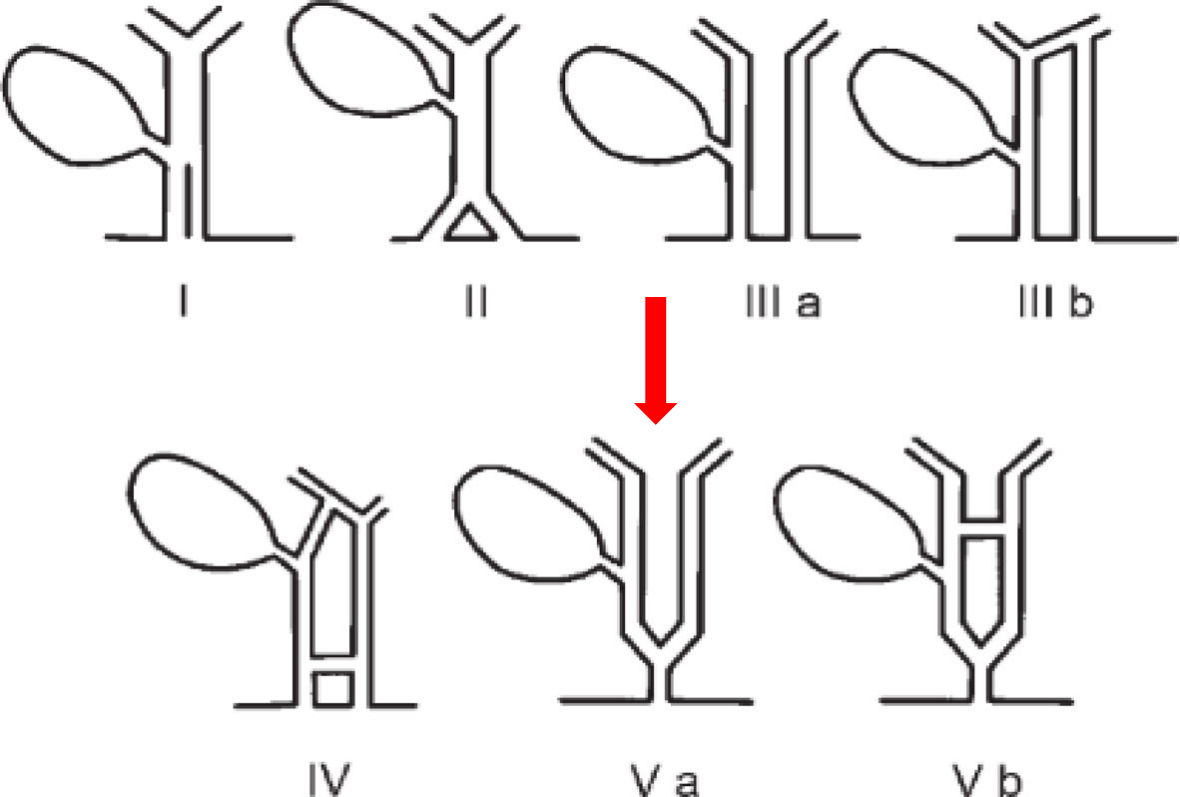
Modified double common bile duct classification proposed by Choi *et al*. [4] (Our case *arrowed*)

Duplication of the extrahepatic bile duct is one of the rarest congenital variants. The first description of this anomaly was done by Vesalius in 1543 [1]. This entity has been sparingly reported, with less than 30 cases reported in the Western literature [2–5]. Most of the cases are reported from Asian countries. Yamashita *et al*. subsequently recorded 47 cases in Japanese literature from 1968 to 2002 [6]. Choi *et al*. classified these anomalies into five different types (Fig. 1) [4]. Among these, type Va has been reported only two times in the literature [4, 7]. We present a case of duplication of the extrahepatic bile duct (Type Va) along with cholelithiasis and choledocholithiasis.

## Case presentation

A 63-year-old woman from rural Nepal presented with a history of right upper abdominal pain, epigastric discomfort, bloating, and dyspepsia for 4 years, followed by intermittent fever and jaundice for a month. She had an increase in severity of pain, yellowish discoloration of body, vomiting, and fever for 1 week but had no history of anorexia or weight loss. She had no significant past medical and surgical history. On general examination she had icterus. Her vital parameters were within the normal range. An examination of her abdomen revealed a non-distended soft abdomen with negative Murphy’s sign and her gall bladder was not palpable.

**Fig. 2:**
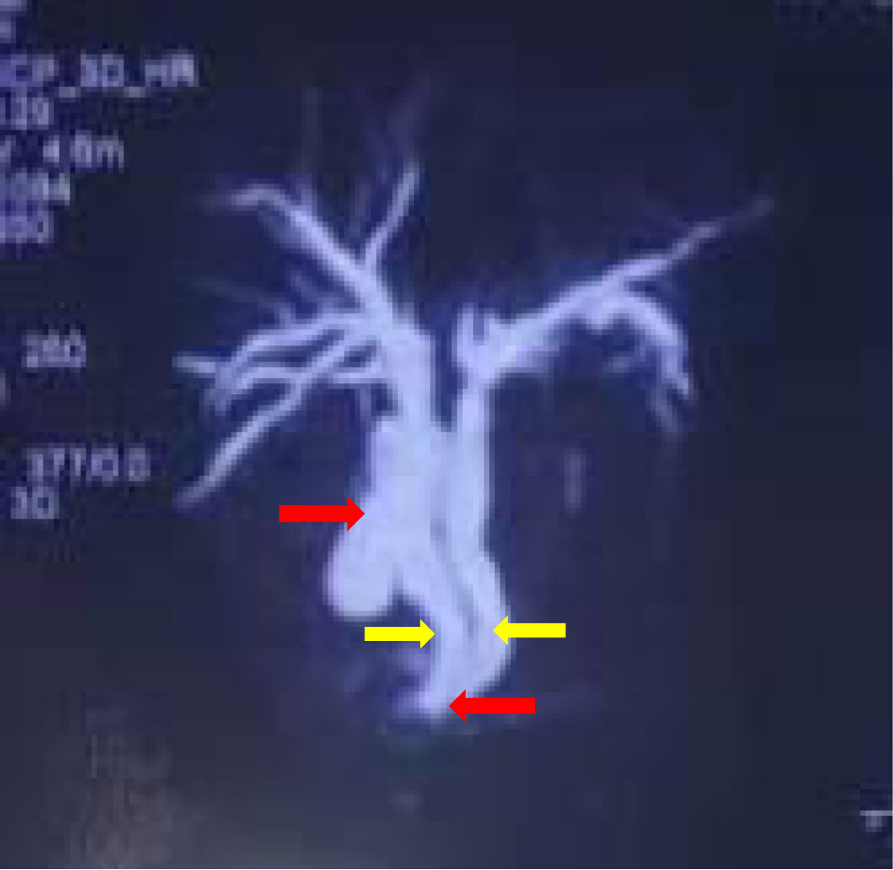
Magnetic resonance cholangiopancreatography. *Right red arrow* – gall bladder draining to right hepatic duct. *Right yellow arrow* – right hepatic duct. *Left yellow arrow* – left hepatic duct. *Left red arrow* – abrupt cutoff point and choledocholithiasis in common bile duct

Biochemical parameters showed hemoglobin 13.4 gm/dl, total leukocyte count 12,600/mm^3^, platelets 158,000/mm^3^, total bilirubin 7.9 mg/dl, direct bilirubin 5.4 mg/dl, alanine aminotransferase (ALT) 196 U/L, aspartate aminotransferase (AST) 146 U/L, alkaline phosphatase (ALP) 273 U/L, serum amylase 50 U/L, and lipase 100 U/L. An abdominal ultrasonogram showed multiple calculi in her gallbladder (largest 6 mm), dilated common bile duct (CBD) measuring 13 mm with dilated intrahepatic biliary ducts (IHBDs), and suspected calculi/mass in distal CBD. A contrast-enhanced computed tomography (CECT) scan of her abdomen revealed similar findings and could not differentiate mass or stone in distal CBD. Endoscopic retrograde cholangiopancreatography (ERCP) was planned with the aim of extracting the stone and, if present, a biopsy from the mass. ERCP was unsuccessful on account of difficult anatomy. Magnetic resonance cholangiopancreatography (MRCP) reported the following: IHBDs from the right lobe of the liver drained into the right hepatic duct that formed a separate CBD with the cystic duct opening into it; the IHBDs from the left lobe of the liver drained into the left hepatic duct that formed a separate CBD. Both CBDs appeared mildly dilated proximally and descended separately until a point where a stone was noted. Below this point, the CBD could not be traced so the type of anomaly was inconclusive (Fig. 2).

**Fig. 3:**
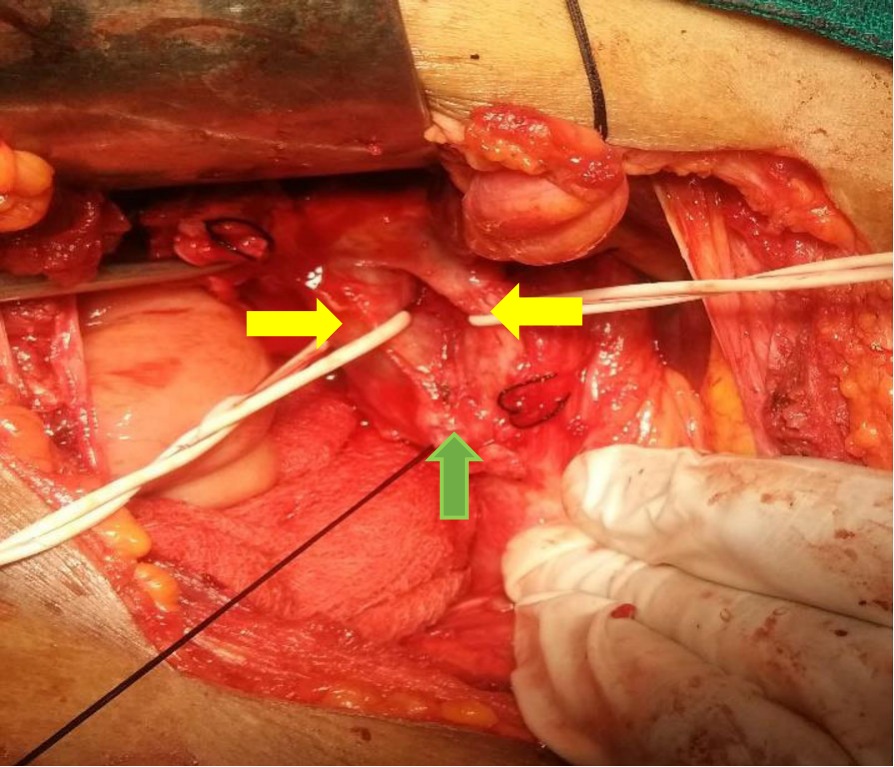
Intraoperative photograph. *Right yellow arrow* – right hepatic duct. *Left yellow arrow* – left hepatic duct. *Green arrow* – joining of two hepatic ducts to form common bile duct

Due to radiological findings that were suspicious of double CBD (DCBD), our patient was then planned for exploratory laparotomy to assess and confirm the radiological findings, and for definitive diagnosis and treatment of the condition. Intraoperative findings were contracted gall bladder with multiple tiny calculi, two separate draining bile ducts, which were fused just before opening to the ampulla, common duct with a calculus of 1 × 1 cm^2^, and cystic duct opening into the right duct (Fig. 3). So the diagnosis of type Va DCBD with cholelithiasis and choledocholithiasis was made. Following this, cholecystectomy was done, the common duct was explored and the stone was extracted. The right duct was divided just proximal to the opening of the cystic duct and the left duct was also divided at the same level. After closing distal common bile duct stump, reconstruction was done by hepatic ductoplasty (joining two ducts) with hepaticojejunostomy in Roux-en-Y fashion. Her postoperative period was uneventful and she was discharged after 1 week and was doing well at 6-month follow-up.

Histologic examination of the resected specimens revealed chronic inflammatory changes in the gallbladder and common bile duct with no evidence of malignancy.

## Discussion

In the literature “common bile duct” is defined as a duct that directly drains into the gastrointestinal tract irrespective of its proximal anatomy [4]. Our case consists of two long extrahepatic bile ducts joined distally to form a single short common bile duct with the cystic duct draining into the right hepatic duct without the presence of a common hepatic duct. Mechanisms involved in this developmental anomaly include disturbance in the recanalization of the hepatic primordium, random subdivision of the hepatic diverticulum during the first week of embryogenesis, and early disruption of the development of CBD, resulting in the persistence of an extrahepatic accessory duct that is present in early embryogenesis and regresses with normal development [4, 8, 9]. The first description of DCBD was done by Vesalius in 1543 [1]. It is a very rare anomaly, since Western studies have reported less than 30 cases from 1543 to 2007 [2–5]. On the other hand, Yamashita *et al*. reviewed Japanese literature from 1968 to 2002 and found 47 patients with this anomaly [6]. The first classification of this anomaly was done by Goor and Ebert (1972) [10], which was subsequently modified by Saito *et al.* (1988) [11] and recently by Choi *et al*. (2007) [4] (Fig. 1). The individual subtypes are as follows: type I, a CBD with a septum in the lumen; type II, a CBD that bifurcates and drains separately; type III, double biliary drainage without extrahepatic communicating channels, that is, without (a) or with intrahepatic communicating channels (b); type IV, double biliary drainage with one or more extrahepatic communicating channels; and, type V, single biliary drainage of double extrahepatic bile ducts without (a) or with communicating channels (b). Most commonly reported are types III and IV [12]. The least common is type V with only two cases similar to ours of type Va and one case of Vb reported in the literature to date [4, 7, 13]. This anomaly is clinically important as it may be associated with anomalous pancraticobiliary maljunction (PBM) (29%) or complications like: gallstones and choledocholithiasis (27%) as in our case; cancers of the upper gastrointestinal tract, pancreas, and biliary system (25%); and choledochal cyst (10%) [4, 6, 12]. Gastric cancer is seen most commonly when an accessory duct drains into the stomach (as in type III variant) while pancreaticobiliary malignancies are more often seen when the accessory duct opens into the duodenum through the major papilla and is associated with PBM [6]. These associated conditions determine the treatment and prognosis. The preoperative diagnosis of this rare anomaly becomes more vital due to the risk of iatrogenic injury to the extrahepatic bile ducts. ERCP is regarded as the gold standard; however, due to difficult anatomy it may not be possible to cannulate in some patients, multidetector computed tomography (CT) and MRCP is a well-established excellent non-invasive alternative that provides analogous anatomic information of the pancreaticobiliary ducts [13–15]. It is not always possible to diagnose definitive biliary disorders preoperatively with these techniques. Most patients with bile duct duplications undergo surgery because of complications arising from the anomaly. Patients with severe symptoms and complications may need surgical treatment as in our case; our patient developed cholelithiasis, choledocholithiasis, and recurrent cholangitis.

## Conclusion

A rare case of DCBD (type Va) complicated by choledocholithiasis, cholangitis, and chronic cholecystitis is reported here. Precise preoperative recognition is crucial. MRCP and multidetector CT are helpful in making the diagnosis. The case exemplifies how rare cases are overlooked by modern diagnostic techniques. Correct diagnosis helps appropriate surgical intervention.

## Data Availability

All data and materials are available for sharing if needed.
